# Increasing the Bioactive Compound Content of Olive Oil by Acidification of Olive Paste

**DOI:** 10.3390/foods14081336

**Published:** 2025-04-12

**Authors:** Raúl Peralta, Alfonso M. Vidal, Francisco Espínola, María Teresa Ocaña, Manuel Moya

**Affiliations:** 1 Chemical, Environmental and Materials Engineering Department, University of Jaén, Paraje Las Lagunillas, 23071 Jaén, Spain; rpgarcia@ujaen.es (R.P.); amvidal@ujaen.es (A.M.V.); fespino@ujaen.es (F.E.); 2 Institute of Biorefineries Research (I3B), University of Jaén, Campus Las Lagunillas, 23071 Jaén, Spain; 3 Science Didactics Department, University of Jaén, Paraje Las Lagunillas, 23071 Jaén, Spain; mocana@ujaen.es

**Keywords:** oleacein, oleocanthal, hydroxytyrosol, secoiridoid, antioxidant capacity, phenolic compounds

## Abstract

This study investigated the impact of acidification on olive paste to enhance the extraction of olive oil enriched in bioactive phenolic compounds, aiming to develop a novel functional food. Recognizing that acidic pH promotes the activity of *β-glucosidase* responsible for oleuropein and ligstroside hydrolysis, food-grade organic acids—citric, ascorbic, and acetic acid—were added prior to malaxation to improve the bioactive compound content in the resulting oils. A randomized experimental design was employed, using three different doses of each acid (1, 2, and 4%) with three replicates per dose, alongside control trials without acid addition. Acidification did not affect olive oil extraction efficiency or alter quality parameters for extra virgin olive oils. Treatment with 1% ascorbic acid significantly increased phenolic compound content by 37% compared to controls. Secoiridoids comprised 79.4% of the total phenolic content, with oleacein as the predominant compound (237.58 ± 9.18 mg/kg), representing over 50% of the total. This increased oleacein concentration led to up to a 67% enhancement in antioxidant capacity (428.63 ± 31.57 mg Trolox/kg) compared to controls. The antioxidant capacities of 3,4-DHPEA, 3,4-DHPEA-EDA, and 3,4-DHPEA-EA were determined to be 12.05, 5.80, and 4.91 µmol TE/mg, respectively. Furthermore, the 1% ascorbic acid treatment enhanced volatile compounds associated with the lipoxygenase (LOX) pathway while reducing ethanol levels.

## 1. Introduction

Olive oil is highly valued for its exceptional nutritional properties and associated health benefits. It is widely consumed globally, playing a particularly crucial role in Mediterranean countries, which account for a substantial portion of global olive oil production and consumption [1]. Its composition, characterized by unsaturated fatty acids, bioactive compounds, and a distinctive flavor and aroma profile, makes it a unique dietary fat [2].

Several factors influence the phenolic composition and quality of olive oil, including geographical origin, soil composition, climatic conditions, agricultural practices, olive cultivar, fruit ripeness, and processing parameters [3]. Among the minor components in olive oil, phenolic compounds are crucial due to their antioxidant properties and their contribution to the stability, bitterness, and pungency of virgin olive oils. These bioactive molecules have demonstrated potential in mitigating inflammation, cardiovascular diseases, certain cancers, microbial infections, and cellular oxidative stress [4,5,6,7,8,9]. The European Commission has authorized a health claim for olive oil, stating that ‘olive oil polyphenols contribute to the protection of blood lipids against oxidative damage’. This claim is valid provided a minimum daily intake of 20 g of olive oil containing at least 250 mg/kg of polyphenols, particularly hydroxytyrosol and its derivatives, is consumed [10].

**Figure 1:**
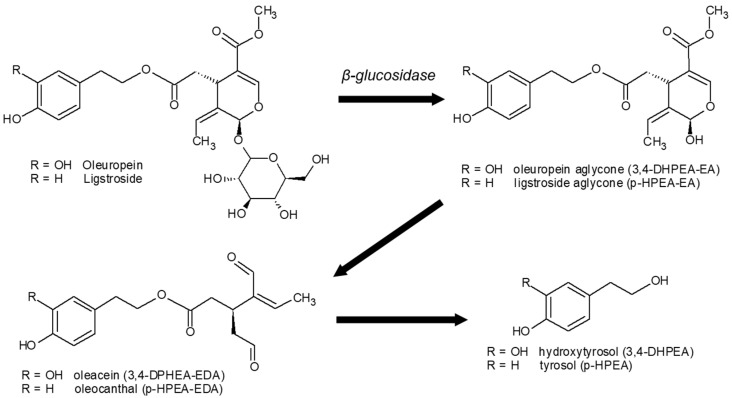
Secoiridoid formation from oleuropein/ligstroside hydrolysis and the subsequent conversion to hydroxytyrosol/tyrosol.

Secoiridoids constitute the most abundant group of phenolic compounds in olive oil, comprising approximately 80% of total phenolics. These compounds are formed during oil extraction through the hydrolysis of oleuropein and ligstroside. Oleacein (3,4-DHPEA-EDA) and oleocanthal (*p*-HPEA-EDA) are the predominant secoiridoids, while their precursor aglycones (3,4-DHPEA-EA and *p*-HPEA-EA) complete this group [11]. Hydroxytyrosol and tyrosol are the final decomposition products of oleacein and oleocanthal, respectively (Figure 1). Among all antioxidants present in olive oil, hydroxytyrosol (3,4-DHPEA) and its secoiridoid derivatives (3,4-DHPEA-EDA and 3,4-DHPEA-EA) are considered the most potent, exhibiting greater antioxidant activity than even α-tocopherol [3]. Other phenolic constituents of virgin olive oil include phenolic acids (e.g., vanillic, ferulic, and *p*-coumaric acid), lignans (such as pinoresinol), and flavonoids (such as luteolin and apigenin) [12,13,14].

The phenolic and volatile profiles of olive oil are largely determined by the enzymatic activity of the olive fruit, which is influenced by factors such as ripening stage, geographical origin, and water stress. Furthermore, the technological conditions employed during oil extraction significantly affect these profiles [15,16,17,18,19]. In particular, the milling and malaxation steps play a crucial role in regulating the secoiridoid content of olive oil. Lower milling temperatures and controlled malaxation conditions have been associated with higher retention of these valuable phenolic compounds. Optimal conditions include malaxation at temperatures between 25 and 27 °C, acidic pH, and minimal oxygen exposure, as these conditions favor the enzymatic activity of endogenous lipoxygenase and *β-glucosidase*, which contribute to higher concentrations of volatile compounds and secoiridoids [20,21,22]. Conversely, increased olive ripeness leads to the activation of phenol oxidase enzymes, reducing the levels of oleacein, oleocanthal, and their derivatives.

Oleocanthal-rich oils have been linked to improved cognitive function in patients with Alzheimer’s disease [23]. Additionally, oleocanthal offers cardiovascular benefits and has demonstrated therapeutic potential for treating inflammation, cancer, and neurodegenerative conditions [24,25]. Similarly, oleacein exhibits antioxidant and anti-inflammatory properties, making it valuable in addressing inflammatory diseases and hypertension [26,27]. Due to the multiple health benefits of these secoiridoids, numerous studies have attempted to isolate and concentrate oleacein and oleocanthal from olive oil using various extraction methods [28,29,30,31].

The aim of this study is to produce olive oils with a high content of bioactive phenolic compounds. Olive oils obtained through this process can be marketed as functional foods, offering enhanced health benefits due to the bioactive properties of these phenolic compounds. To achieve this, food-grade organic acids—citric, ascorbic, and acetic acids—will be used to modify enzymatic activity, directing metabolic pathways toward maximizing the content of the desired oleuropein/ligstroside derivatives. Additionally, this study will determine which acid and its optimal concentration yield the best results in terms of extraction efficiency, volatile compound profile, antioxidant capacity, individual secoiridoid content, total secoiridoid concentration, and overall phenolic content.

Ascorbic acid, also known as vitamin C, is a water-soluble organic compound that is insoluble in fats and oils. It is classified as a food additive (E300) and exhibits antioxidant properties. Due to its high antioxidant capacity, vitamin C is commonly used in aqueous solutions, oil-in-water emulsions, and oleogels; however, it is not soluble in refined oils or oils devoid of moisture [32,33,34]. Citric acid is an organic compound predominantly found in citrus fruits and is responsible for their sour taste. It is water-soluble but insoluble in oils and fats. Although its antioxidant capacity is lower than that of α-tocopherol, it promotes the synthesis of antioxidant compounds in the body [35]. When added to olive paste, it has been shown to increase the phenolic compound content of the resulting oils by up to 36% [36]. It is classified as a food additive (E330) and is commonly used as an acidulant and flavoring agent. Acetic acid is an organic compound formed through the oxidation of ethanol and is responsible for the characteristic sour taste of vinegar. It is soluble in water but only sparingly soluble in oils and fats. Unlike ascorbic and citric acids, acetic acid does not exhibit direct antioxidant properties. It is classified as food additive E260 and is primarily used as an acidulant and preservative.

## 2. Materials and Methods

### 2.1. Olive Characterization

Olives (*Olea europaea* L. cv. Picual) were hand-harvested from rainfed orchards in Jaén, southern Spain, during the 2023–2024 crop season. The maturity index of the olives was 1.4, determined according to the method described by Vidal et al. [31]. Moisture content was measured by drying the milled olive paste at 105 °C for 24 h. Oil content was determined using Soxhlet extraction, following Commission Regulation guidelines with prior modifications [10]. For oil extraction, approximately 10 g of the sample were extracted for 4 h using hexane.

### 2.2. Oil Extraction

The olive samples were extracted using a laboratory-scale Abencor system (MC2 Ingeniería y Sistemas S.L., Sevilla, Spain), as described by Vidal et al. [31], which includes malaxation in open chambers. The extraction conditions for all experiments were set to 500 g of olives, a malaxation time of 60 min, a malaxation temperature of 25 °C, and a crusher hole diameter of 5.5 mm. These parameters were selected based on previous research, which identified them as optimal for achieving industrially viable yields while maintaining a balanced composition of phenolic and volatile compounds. The low malaxation temperature was specifically chosen to favor lipoxygenase and *β-glucosidase* enzymatic activity [37].

Prior to malaxation, appropriate doses of ascorbic acid (asc) (ascorbic acid, analytical grade, Panreac, purity 99.0%), citric acid (cit) (citric acid anhydrous, pharmaceutical grade, Panreac, purity 99.5%), and acetic acid (act) (acetic acid glacial, analytical grade, Panreac, purity 99.7%) were incorporated into the olive paste. Each acid was added at concentrations of 1, 2, and 4% (*w*/*w*) to independent olive paste samples, with three replicates per condition. The ascorbic and citric acids were added in powder form, consistent with their commercial presentation, while glacial acetic acid was added in liquid form. A control sample (ctr) was also prepared without acid addition. Sample testing, including replicates, was conducted in random order (Supplementary Material Table S1).

To maximize extraction, the malaxed pastes were subjected to a two-stage centrifugation process, each stage lasting 180 s. The resulting supernatant liquids were collected in graduated cylinders. Following extraction, the oil underwent a 24 h decantation period, was filtered through cellulose paper to remove organic matter and moisture and was stored under a nitrogen atmosphere at −18 °C until analysis. Extraction efficiency was calculated as the percentage of oil extracted relative to the actual oil content in the drupes, as defined by Vidal et al. [31].

### 2.3. Analysis of Olive Oil Quality Parameters

The peroxide index, free acidity, and extinction coefficient K_270_ were measured following the guidelines established by the Commission Regulation [38].

### 2.4. Phenolic Compounds

Sample treatment was conducted according to the methodology proposed by the International Olive Council (IOC) [39]. Details of the analytical method used were previously described in Vidal et al. [37].

Phenolic compound quantification was performed using High-Performance Liquid Chromatography (HPLC) (Shimadzu Corp., Kyoto, Japan), equipped with a BDS Hypersil C18 column (Thermo Scientific, Waltham, MA, USA). The column specifications were 25 cm in length, a 5 μm particle size, and a 4.6 mm internal diameter. The mobile phase consisted of water with 0.2% orthophosphoric acid, methanol, and acetonitrile. The elution flow rate was set at 1 mL/min, the oven temperature at 30 °C, and the sample injection volume at 20 μL.

Phenolic compounds were identified by comparison with analytical standards, except for ligstroside aglycone and unidentified phenols, whose concentrations were determined according to the IOC method. Results are expressed as milligrams of compound per kilogram of oil.

The following standards were used for phenolic compound identification: Vanillic acid, vanillin, luteolin, and *p*-coumaric acid (purchased from Sigma-Aldrich, St. Louis, MO, USA); Syringic acid and *trans*-ferulic acid (purchased from Fluka, Milan, Italy); hydroxytyrosol, tyrosol, and apigenin (purchased from HPC Standards GmbH, Cunnesdorf, Germany); oleocanthal (purchased from Phytolab, Dutendorfer, Germany); oleacein, oleuropein aglycone, and pinoresinol (purchased from TRC, Toronto, ON, Canada).

### 2.5. Volatile Compounds

The determination of volatile compounds was carried out using the method previously described by Vidal et al. [31], which employs headspace solid-phase microextraction (HS-SPME) coupled with gas chromatography-flame ionization detection (GC-FID). The analyses were performed using a 7890B model gas chromatograph equipped with a DB-WAXetr capillary column (Agilent Technologies, Santa Clara, CA, USA). The SPME fiber, composed of Carboxen/DVB/polydimethylsiloxane (2 cm in length, 50/30 μm film thickness), was obtained from Supelco (Bellefonte, PA, USA). 4-Methyl-2-pentanol was used as the internal standard, and 35 external standards were employed for compound identification.

The analyzed volatile compounds were categorized into three groups: C6 LOX-derived compounds: Hexanal, hexanol, (E)-2-hexenal, (E)-2-hexenol, (Z)-3-hexenol, and (Z)-3-hexenyl acetate; C5 LOX-derived compounds: 1-Penten-3-ol, 1-penten-3-one, and (Z)-2-pentenol; and ethanol. Results are expressed as milligrams of compound per kilogram of olive oil.

### 2.6. Antioxidant Capacity

The antioxidant capacity was assessed using the ferric ion reducing antioxidant power (FRAP) method, following the protocol described in Vidal et al. [40]. A Trolox standard curve was used for calibration, and the results are expressed as Trolox equivalents (TE) per kilogram of oil. Among the various methods for antioxidant analysis, the FRAP method was chosen to examine the dependence of the phenolic antioxidant content in oils on the acids and their concentrations, as it provides the most reproducible results with minimal experimental errors.

### 2.7. Statistical Analysis

Data were statistically analyzed using StatGraphics Centurion 19.1.2 (Statpoint Technologies, Inc., Warrenton, VA, USA). Fisher’s least significant difference (LSD) test was applied to evaluate statistical differences among the responses analyzed.

## 3. Results

### 3.1. Characterization of Olive Fruit

Olive fruit samples, utilized as experimental raw material, were analyzed in quadruplicate. Mean values and standard deviations for moisture, oil, and solid content are presented in Table 1.

### 3.2. Extraction Efficiency and Water pH

Table 2 presents the mean extraction efficiencies and water pH values post-oil extraction, accompanied by their respective standard deviations. Statistically significant differences between mean values within each column are denoted by distinct superscript letters. Statistical differences were determined using analysis of variance (ANOVA) and a post hoc multiple range test based on Fisher’s least significant difference (LSD) at a significance level of *p* < 0.05.

### 3.3. Oil Quality Parameters

Although the extracted oils cannot be classified as virgin olive oils under Regulation (EU) 1308/2013 [41], due to the use of processing aids with a chemical action that lowers the pH of the medium, the most common quality parameters were analyzed to assess whether acid addition affects the extracted oils. Free acidity was measured in all oil samples except those obtained from pastes treated with acetic acid, because acetic acid content is considered a negative quality parameter according to the International Olive Council (IOC) [42]. Among the oils for which free acidity was determined, no statistically significant differences were observed between the control samples and those treated with acids. The mean free acidity was 0.14 ± 0.02% (g oleic acid per 100 g of olive oil). Similarly, no statistically significant differences were detected for the peroxide index or the K_270_ extinction coefficient. The peroxide index averaged 2.35 ± 0.32 mEq O_2_/kg, while the K_270_ value was 0.16 ± 0.02. These findings indicate that the presence of acids does not alter the main quality parameters of the extracted oils. Based on these results, the oils obtained could be classified as extra virgin according to conventional quality parameters.

### 3.4. Phenolic Compounds and Antioxidant Capacity

All the phenolic compound content was determined by HPLC. The phenolic alcohol, flavonoid, and lignan contents in oils are shown in Table 3. Phenolic acids and their derivatives are presented in Table S2. The results are displayed as means ± standard deviations from three independent assays (n = 3), with statistical significance (*p* < 0.05) between means indicated by letters based on Fisher’s LSD.

Table 4 presents the secoiridoid and total phenolic contents, along with the antioxidant capacity of the olive oils, as determined by the FRAP method. All secoiridoids originate from the hydrolysis of oleuropein and ligstroside. Among these compounds, oleuropein derivatives were the most abundant across all produced oils, particularly oleacein (3,4-DHPEA-EDA) and its precursor, oleuropein aglycone (3,4-DHPEA-EA). Ligstroside derivatives, including oleocanthal (*p*-HPEA-EDA) and its aglycone (*p*-HPEA-EA), were present at lower concentrations. Like the prior tables, Table 4 incorporates statistical analysis, using superscript letters to differentiate means within each column based on Fisher’s LSD (*p* < 0.05).

### 3.5. Volatile Compounds and Ethanol Content

Table 5 shows the effect of olive paste acidification on the concentration of volatile compounds associated with the lipoxygenase (LOX) pathway, specifically those with five (C5) and six (C6) carbon atoms, and ethanol, an undesirable compound linked to the fermentation of organic residues in olive oil.

## 4. Discussion

### 4.1. Extraction Efficiency and Water pH

The statistical analysis presented in Table 2 demonstrated that neither the type of acid nor its dosage significantly influenced extraction efficiency (*p* = 0.4, α = 0.05). Although the mean extraction efficiency for pastes treated with 1% ascorbic acid (68.83%) was slightly elevated compared to the control (67.81%), this observed difference did not achieve statistical significance. Consequently, the results indicate that the application of acids, within the tested parameters, did not produce a statistically significant change in olive paste extraction efficiency. These results confirm that the acids did not have direct technological effects. The primary objective of this study was to enhance *β-glucosidase* activity, thereby increasing bioactive phenolic compound concentrations, by utilizing the enzyme’s known preference for acidic pH and relatively low temperatures.

As for pH in Table 2, the pH of the vegetation waters from extracted pastes exhibited a decrease with increasing acid concentration. Notably, the highest acid concentrations resulted in the most significant pH reductions. It is important to note that acid concentrations were expressed as a percentage of paste weight rather than as molar ratios. Because the molecular masses of the acids differ—acetic acid (60.05 g/mol), ascorbic acid (176.12 g/mol), and citric acid (192.12 g/mol)—proportionally more acetic acid was added than ascorbic or citric acid at the same percentage. This, combined with the differences in pKa values (acetic acid: 4.74, ascorbic acid: 4.10, citric acid: 3.15), resulted in water samples from acetic acid treatments being nearly three times more concentrated than those obtained with ascorbic or citric acid at the same weight percentage. Considering these factors, the pH values in Table 2 are justified. Among all treatments, pastes treated with 1% ascorbic acid exhibited the smallest reduction in water pH, decreasing from 4.95 in the control (no acid added) to 4.33 with 1% ascorbic acid.

Consequently, the question arises whether the pH reduction in the pastes, induced by acid addition, may shift the equilibrium of certain phenolic compounds, responsible for the inherent acidity of the control pastes, towards their molecular form, thus enhancing their solubility in the oil phase. These authors posit that the phenolic compound group is diverse, with phenolic acids being prominent constituents that contribute to the inherent acidity of the control pastes. In contrast, secoiridoids are not characterized by high acidity. Their principal functional groups, alcohols, exhibit significantly lower acidity (pKa secoiridoids ≫ pKa phenolic acids). Within olive paste, the dissociation of secoiridoids is further suppressed by the presence of phenolic acids and the prevailing pH, leading to their predominantly molecular form. The subsequent sections will elaborate on the influence of pH.

### 4.2. Phenolic Compounds and Antioxidant Capacity

Table 3 demonstrates that acid treatments generally elevated hydroxytyrosol levels. Specifically, increasing concentrations of acetic and citric acids resulted in progressively higher hydroxytyrosol content, with the ‘High’ concentration treatments approaching a doubling of the control value (2.20 ± 0.21 mg/kg). Initially, it might be hypothesized that a reduction in paste pH promotes hydroxytyrosol formation, thereby enhancing its partitioning into the oil phase via equilibrium. However, correlation analysis between hydroxytyrosol content and pH revealed no significant trend, indicating that pH is not the primary determinant of hydroxytyrosol enrichment in the oils. Notably, ascorbic acid at the ‘Low’ concentration yielded the highest hydroxytyrosol level among all treatments (3.03 ± 0.35 mg/kg), suggesting a distinct mechanism of action.

Regarding tyrosol, acetic and citric acids exhibited a dose-dependent increase in oil concentration, whereas ascorbic acid resulted in a reduction, independent of the applied dose.

An alternative method for obtaining oils rich in specific bioactive compounds, employing supercritical CO_2_ extraction, yielded hydroxytyrosol and tyrosol concentrations of 13 and 29 mg/kg, respectively [43]—markedly higher than our findings. In a study comparing pharmacologically relevant phenolic compounds in various Omani olive oil cultivars, the Picual variety exhibited mean values (mg/kg) of approximately 4.2 for hydroxytyrosol and 1.6 for tyrosol [44]. Notably, the hydroxytyrosol content in our oils produced with the highest citric and acetic acid doses exceeded this value, and several of our acidified conditions also demonstrated higher tyrosol concentrations.

In the flavonoid and lignan columns of Table 3, luteolin is shown to be the predominant flavonoid in all oils extracted from acidified pastes. However, its concentration only increased compared to control oils when ascorbic acid was applied. On the contrary, the apigenin content increased with all acids, with the most significant increase being observed at low ascorbic acid concentrations, tripling the content in the oils with respect to the control. For lignan pinoresinol, all concentrations of citric acid led to an increase compared to the control oils. In contrast, only low concentrations of the other two acids resulted in higher pinoresinol levels than the control oils. In none of the cases did the pH variation due to the different doses of acids seem to have a significant influence on the content of these compounds in the oils.

Table S2 shows the phenolic acids and their derivatives quantified in the oils. Regarding phenolic acids, vanillic acid was identified as the most abundant, followed by *p*-coumaric acid. Vanillic acid concentrations were elevated across all acid treatments compared to the control. However, the dose–response varied among acids. Acetic acid exhibited no discernible dose-dependent effect, citric acid showed an inverse relationship between dose and vanillic acid concentration, and ascorbic acid demonstrated a positive correlation. Notably, despite this positive correlation, ascorbic acid resulted in the smallest overall increase in vanillic acid levels.

*p*-Coumaric acid concentrations were elevated in oils treated with all acids, with no significant variation observed across acid types or dosages. Table S2 indicates that acetic and citric acids did not significantly alter *t*-ferulic acid concentrations in the oils. In contrast, ascorbic acid elicited an increase, exhibiting an inverse dose–response relationship, with the highest *t*-ferulic acid concentration, approximately threefold greater than the control, observed at the lowest dose.

Considering the pH of the vegetation waters, these acids are expected to be predominantly in their molecular form, thus exhibiting increased solubility in the oils. However, this expected trend is not consistently observed in the results. Only *t*-ferulic acid shows a notable increase in solubility in the oils, reaching levels nearly three times higher than those in the control oils.

In a study examining the impact of cultivar on olive oil phenolic profiles across 80 selected varieties over two harvest seasons [45], the first season yielded average (mg/kg) values of 1.68 for hydroxytyrosol (max. 4.89), 3.31 for luteolin (max. 6.25), and 1.87 for apigenin (max. 11.53). The second season’s averages were 2.04 for hydroxytyrosol (max. 7.57), 3.72 for luteolin (max. 11.35), and 2.98 for apigenin (max. 19.79). Our findings indicate that several of our experiments achieved or exceeded the maximum phenolic contents observed in the first season for all three compounds, and surpassed the second-season averages, although we did not reach the second-season maximums. These exceptionally high values in the cited study likely resulted from specific, highly favorable conditions.

All secoiridoids are derived from the hydrolysis of oleuropein and ligstroside (Table 4). Among these compounds, oleuropein derivatives were predominant across all oil samples, with oleacein (3,4-DHPEA-EDA) and its precursor, oleuropein aglycone (3,4-DHPEA-EA), being particularly abundant. In contrast, ligstroside derivatives, including oleocanthal (*p*-HPEA-EDA) and its aglycone (*p*-HPEA-EA), were detected at significantly lower concentrations.

Table 4 shows that the oleacein content is higher in all acid-treated oils compared to the control, especially in the oils obtained from pastes treated with 1% ascorbic acid, where the content is the highest among all treatments, nearly doubling that of the control. The oleuropein aglycone content, however, is lower than in the control in all oils except those extracted from pastes with 1% ascorbic acid. Regarding ligstroside derivatives, the content of both oleocanthal and its aglycone is lower than in the control in all oils, except for oleocanthal in samples obtained from pastes treated with 1% ascorbic acid. Table 4 confirms that oils extracted from 1% ascorbic acid pastes have higher oleacein and oleocanthal content than all other oils, including the control samples. As evidenced by the data presented in Table 4, no discernible correlation between secoiridoid compound content and the pH of the vegetation waters was observed.

Table 4 also presents the total phenolic compound content and antioxidant capacity of the oils. Phenolic content generally decreases as acid concentration increases, with the exception of citric acid, which shows no significant effect. At lower acid concentrations, a higher phenolic content was observed compared to the control oils, particularly in oils extracted using 1% ascorbic acid, which correlates with the elevated secoiridoid levels in these samples. The antioxidant capacity trend mirrors that of oleacein, peaking in oils produced with 1% ascorbic acid. The oils obtained with 1% ascorbic acid exhibit the highest phenolic compound content and antioxidant capacity, 37.2% and 66.9% greater, respectively, than the control oils. Regarding the phenolic compounds discussed previously, neither the total phenolic content nor the antioxidant capacity exhibited a statistically significant correlation with the pH of the vegetation waters. Consequently, it is inferred that the observed variations in phenolic compounds within the oils are not attributable to pH, but rather to other factors beyond the scope of this study.

Based on the experimental results, it is concluded that the acids introduced into the pastes do not function as technological coadjuvants, as they fail to alter extraction yields or other process-dependent oil parameters. However, they can be categorized as chemical coadjuvants due to their ability to modify the paste pH and, critically, to enhance the *β-glucosidase*-mediated hydrolysis of oleuropein and ligstroside into their respective derivatives, thereby indirectly enriching the oils with secoiridoid compounds, which is the primary objective of this study.

The mechanism by which low-dose ascorbic acid enhances secoiridoid content in oils remains unclear, with a paucity of supporting literature. We hypothesize that, in addition to pH reduction and consequent facilitation of *β-glucosidase* activity, ascorbic acid may modulate the antioxidant capacity, thereby attenuating phenol oxidase activity. This attenuation could mitigate the conversion of oleuropein to oxidized forms that are not the target compounds of this study.

Due to the diversity of agronomic, meteorological, and technological factors that influence the composition of olive oil, no two oil samples are exactly alike [3,46]. Therefore, it is not possible to claim that one olive oil inherently contains more or fewer phenolic compounds than another, as their composition is highly dependent on external factors. Rather, it is only feasible to determine whether a given factor influences a particular parameter or response when tested on a single, standardized olive sample. Consequently, this study exclusively manipulated the type and dosage of acid, utilizing a single olive sample across all trials, while maintaining all other process parameters constant to isolate the impact on the responses under investigation.

Presti et al. [7] analyzed 51 Italian and Spanish extra virgin olive oils (EVOOs), reporting total phenolic contents ranging from 29.5 to 316.3 mg/kg, depending on the cultivar, values lower than those found in this study. López-Huertas et al. [47], using the Picual cultivar, investigated oils processed at temperatures below 27 °C with 30 min malaxation times, reporting a maximum phenolic content of 296 mg/kg at the optimal fruit ripening stage. Similarly, Tomé-Rodríguez et al. [46] analyzed the phenolic profile of virgin olive oil (VOO) from 40 cultivars across three growing seasons, emphasizing the impact of fruit moisture on phenolic content. For the Picual cultivar, average oleacein and oleocanthal contents were reported as 97.3 and 67.1 mg/kg, respectively. Some oils produced in this study through acidification exceeded these reported values by more than 100%.

Oman-grown Picual olives yield oils with average oleacein and oleocanthal concentrations of 43.5 and 58.4 mg/kg, respectively (total: 101.9 mg/kg) [44]. Our control oils demonstrated a combined average of 212.1 mg/kg for these two compounds, and the 1% ascorbic acid-treated oils reached an impressive 329.0 mg/kg. This represents a remarkable increase over the Omani Picual variety (more than threefold) and approximately doubles the reported values for phenolic-rich cultivars like Coratina (181.2 mg/kg) and Kalamata (137.2 mg/kg).

In contrast, a study examining the impact of adding fresh leaves from the Arbequina and Santulhana varieties during oil extraction reported a reduction in phenolic content, attributing this to the partial inhibition of *β-glucosidase* [48]. This finding contradicts another study, which, using Arbequina leaves, demonstrated the effectiveness of this method in enhancing phenolic content [49].

From the data in Table 4, the total secoiridoid content in the oils can be determined. Comparing this value with the total phenolic compound content reveals that secoiridoids account for more than 75% of the total phenols, with oils obtained using low doses of ascorbic acid exhibiting the highest secoiridoid percentage (79.4%). It was observed that the trend in antioxidant capacity paralleled the phenolic and secoiridoid content, with the highest values documented at the lowest ascorbic acid concentrations, specifically 1% (Figure S1, Supplementary Material).

**Figure 2:**
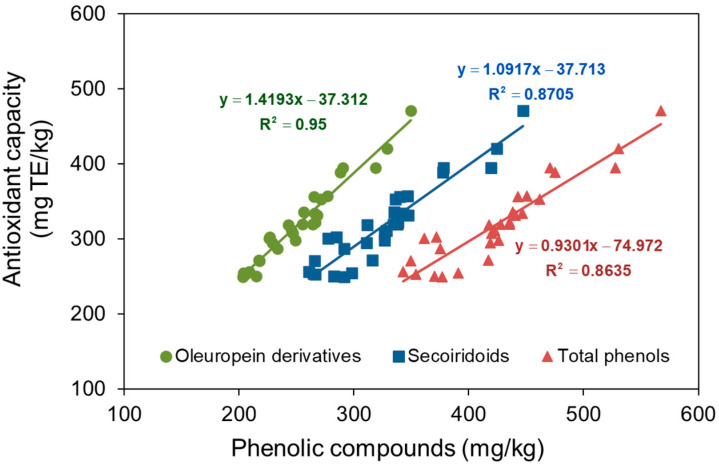
Relationship between antioxidant capacity and the content of oleuropein derivatives (hydroxytyrosol + oleacein + oleuropein aglycone), secoiridoids, and total phenols in the oils.

While minor compounds in olive oil, such as tocopherols, also contribute to its antioxidant capacity, their relatively low concentrations suggest that the variations observed in this study are primarily attributable to changes in the content of oleuropein-derived secoiridoids—3,4-DHPEA-EA and 3,4-DHPEA-EDA—and the final phenolic alcohol, 3,4-DHPEA, as proposed by Servili et al. [3]. Figure 2 illustrates the correlation between antioxidant capacity and the concentrations of oleuropein derivatives (including hydroxytyrosol), secoiridoids, and total phenolic compounds, along with their respective regression lines. The highest coefficient of determination was observed for oleuropein derivatives, followed by secoiridoids, and then total phenolic content. This indicates that oleuropein derivatives exhibit the strongest correlation with antioxidant capacity compared to other phenolic groups. The regression line for oleuropein derivatives suggests that, even in their absence, other antioxidant compounds are present in the oil. Based on the oleacein slope in Figure 2, an estimated antioxidant capacity of 1.42 mg Trolox equivalent per mg oleuropein derivative is obtained (5.67 µmol TE/mg).

**Figure 3:**
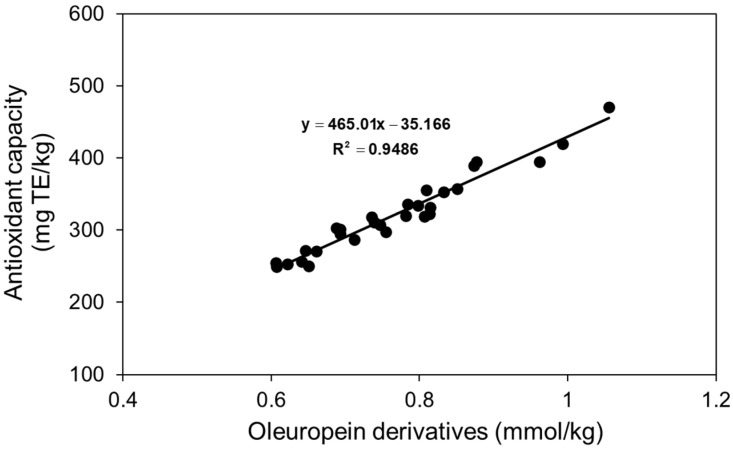
Relationship between antioxidant capacity and the content of oleuropein derivatives (hydroxytyrosol + oleacein + oleuropein aglycone).

Because the three molecules derived from oleuropein have different molecular masses, their antioxidant capacity must be normalized to account for these differences, assuming that antioxidant activity is measured on a per-molecule basis. If each molecule exhibits similar antioxidant activity, their antioxidant potential can be determined by converting mg to mmol and applying a linear regression model, as shown in Figure 3. The results in Figure 3 allow for the calculation of individual antioxidant capacities of 12.05, 5.80, and 4.91 µmol TE/mg for 3,4-DHPEA, 3,4-DHPEA-EDA, and 3,4-DHPEA-EA, respectively. These values are highly comparable to those reported by Vidal et al. [31], who determined an antioxidant capacity of 5.85 µmol TE/mg for oleacein.

Vidal et al. [37] previously reported a total phenolic (TF) content antioxidant capacity of 3.87 μmol TE/mg TF in various oils, a value remarkably consistent with the 3.72 μmol TE/mg TF estimated in Figure 2. Although both studies [31,37] employed the same technological factors (malaxation time and temperature, and mill sieve size) and response surface methodology, their approaches differed due to the use of different olive cultivars. Vidal et al. [37] investigated the influence of olive cultivar (Arbequina, Arbosana, and Koroneiki), irrigation practices (irrigated vs. rainfed), and maturity index within a superintensive cropping system. In contrast, Vidal et al. [31] examined the impact of technological factors on the Picual cultivar in traditional cultivation. Notably, both studies used the DPPH method to assess antioxidant capacity.

### 4.3. Volatile Compound Content in the Oils Obtained with Acids

As shown in Table 5, ascorbic and citric acids enhance the concentration of C6 compounds compared to the control oils, whereas acetic acid does not appear to influence these compounds at low concentrations but reduces them at higher concentrations. Both acetic and citric acids exhibit a dose-dependent reduction in C6 compounds, whereas ascorbic acid does not show a clear dose-dependent effect. The lowest dose of citric acid resulted in a twofold increase in C6 compound content in the oils compared to the control. Focusing on oils obtained with 1% ascorbic acid—which yielded the highest extraction efficiency, oleuropein derivatives content, and antioxidant capacity, the primary objectives of this study—the results indicate an approximate 50% increase in C6 compounds.

For C5 compounds, a similar trend to C6 compounds was observed, with a slight increase when ascorbic acid was applied. The highest C5 concentration was detected at the highest ascorbic acid concentration and the lowest citric acid concentration. Both ascorbic and citric acids slightly reduced ethanol content, while acetic acid led to a more pronounced decrease. In oils extracted with 1% ascorbic acid, the ethanol content was approximately 18% lower than in the control samples. Thus, the incorporation of 1% ascorbic acid into the paste promotes enzymatic pathways responsible for generating desirable volatile compounds while simultaneously reducing the presence of ethanol, an undesirable fermentation byproduct.

As with phenolic compounds, the volatile compound composition of olive oils is highly variable, influenced by both extrinsic and intrinsic factors related to cultivation and processing [50]. Peralta et al. [51], in their study on the Royal cultivar, reported variations in volatile compound concentrations depending on whether the analyzed oil was collected from the decanter outlet or the vertical centrifuge and whether malaxation lasted 15 or 30 min. Their findings closely align with the results obtained in this study.

## 5. Conclusions

The results indicate that the use of acids does not statistically affect the extraction efficiency of olive oil or alter the quality parameters regulated for extra virgin olive oils. However, because of the acidification process, these oils should be classified as functional foods.

Acidification of olive paste generally increases phenolic content. Secoiridoids constitute the predominant phenolic group in all oils, comprising over 75% of the total, and exceeding 79% in those obtained with 1% ascorbic acid. Oleacein content is elevated in all acid-treated oils, with peak concentrations observed in oils extracted with 1% ascorbic acid, nearly doubling control levels. Only oils obtained with the lowest ascorbic acid concentration exhibit increased oleuropein aglycone levels compared to controls. Furthermore, the 1% ascorbic acid treatment yields the highest oleacein and oleocanthal concentrations, along with the greatest antioxidant capacity, a near 67% increase relative to controls. While these oils do not exhibit the highest concentrations of volatile compounds, they demonstrate levels exceeding those of the control oils and a concurrent reduction in ethanol content. Thus, the addition of 1% ascorbic acid results in oils meeting extra virgin olive oil standards, characterized by high phenolic content (particularly secoiridoids), enhanced antioxidant capacity, elevated LOX-related volatile compound levels, and decreased ethanol concentrations.

## Figures and Tables

**Table 1:** Composition of the raw olives *.

Water, %	Oil, %	Solids, %
58.65 ± 0.26	13.35 ± 0.52	27.99 ± 0.72

* Mean and standard deviation of four replicates.

**Table 2:** Water pH values post-oil extraction and extraction efficiency of olive oils *.

Acid	Extraction Efficiency, %	pH
Control (0%)	67.81 ± 1.49 ^ab^	4.95 ± 0.03 ^a^
Acetic (1%)	63.74 ± 3.96 ^ab^	4.22 ± 0.04 ^b^
Acetic (2%)	66.27 ± 1.42 ^ab^	3.99 ± 0.02 ^c^
Acetic (4%)	65.26 ± 2.78 ^ab^	3.79 ± 0.01 ^d^
Ascorbic (1%)	68.83 ± 1.17 ^a^	4.33 ± 0.05 ^b^
Ascorbic (2%)	67.53 ± 1.10 ^ab^	3.96 ± 0.18 ^c^
Ascorbic (4%)	63.70 ± 3.60 ^ab^	3.77 ± 0.00 ^d^
Citric (1%)	66.24 ± 1.29 ^ab^	3.89 ± 0.04 ^cd^
Citric (2%)	63.45 ± 4.09 ^b^	3.48 ± 0.03 ^e^
Citric (4%)	67.63 ± 1.83 ^ab^	3.01 ± 0.02 ^f^
Fisher’s LSD **	5.29	0.14

* Mean and standard deviation of three replicates. ** For each column, superscript letters indicate statistical groupings based on Fisher’s least significant difference (LSD) test. Different letters denote statistically significant differences, while identical letters indicate no significant difference at the 95% confidence level.

**Table 3:** Phenolic alcohol, flavonoid, and lignan contents in oils, mg/kg *.

Acid	Hydroxytyrosol	Tyrosol	Apigenin	Luteolin	Pinoresinol
Control (0%)	2.20 ± 0.21 ^g^	1.60 ± 0.20 ^c^	1.64 ± 0.23 ^f^	4.33 ± 0.30 ^b^	17.85 ± 0.66 ^abcd^
Acetic (1%)	2.60 ± 0.07 ^fg^	1.39 ± 0.09 ^cd^	2.12 ± 0.20 ^ef^	3.67 ± 0.30 ^bc^	18.68 ± 0.30 ^abc^
Acetic (2%)	3.46 ± 0.13 ^cd^	1.62 ± 0.17 ^c^	2.55 ± 0.33 ^de^	3.36 ± 0.23 ^c^	16.43 ± 1.07 ^cd^
Acetic (4%)	5.13 ± 0.40 ^a^	2.86 ± 0.37 ^a^	2.59 ± 0.21 ^cde^	3.86 ± 0.35 ^bc^	15.85 ± 1.82 ^d^
Ascorbic (1%)	3.03 ± 0.35 ^def^	0.86 ± 0.10 ^e^	4.97 ± 0.41 ^a^	5.21 ± 0.40 ^a^	19.41 ± 1.22 ^ab^
Ascorbic (2%)	3.18 ± 0.06 ^de^	0.77 ± 0.06 ^e^	3.23 ± 0.41 ^c^	5.28 ± 0.31 ^a^	17.14 ± 0.41 ^bcd^
Ascorbic (4%)	3.91 ± 0.29 ^c^	1.00 ± 0.07 ^de^	2.70 ± 0.35 ^cde^	5.93 ± 0.73 ^a^	17.54 ± 0.28 ^abcd^
Citric (1%)	2.83 ± 0.13 ^ef^	2.19 ± 0.13 ^b^	2.92 ± 0.16 ^cd^	3.79 ± 0.28 ^bc^	18.61 ± 0.87 ^abc^
Citric (2%)	3.00 ± 0.156 ^def^	2.68 ± 0.26 ^a^	2.57 ± 0.25 ^de^	3.76 ± 0.31 ^bc^	19.40 ± 0.81 ^ab^
Citric (4%)	4.55 ± 0.12 ^b^	2.56 ± 0.09 ^ab^	4.28 ± 0.41 ^b^	5.44 ± 0.64 ^a^	19.54 ± 1.99 ^a^
Fisher’s LSD **	0.46	0.39	0.65	0.84	2.36

* Mean and standard deviation of three replicates. ** For each column, superscript letters indicate statistical groupings based on Fisher’s least significant difference (LSD) test. Different letters denote statistically significant differences, while identical letters indicate no significant difference at the 95% confidence level.

**Table 4:** Individual secoiridoid content, total phenolic content, and antioxidant capacity (FRAP) of oils *.

Acid	Oleacein, mg/kg	Oleocanthal, mg/kg	3,4-DHPEA-EA, mg/kg	*p*-HPEA-EA, mg/kg	Total Phenols, mg/kg	FRAP, mg TE/kg
Control (0%)	127.97 ± 2.52 ^e^	84.16 ± 4.95 ^ab^	77.92 ± 4.72 ^b^	11.83 ± 1.06 ^a^	394.92 ± 16.79 ^d^	258.38 ± 9.52 ^e^
Acetic (1%)	173.04 ± 5.10 ^c^	70.61 ± 4.70 ^cd^	75.80 ± 8.04 ^bc^	8.15 ± 1.03 ^cd^	428.75 ± 12.68 ^c^	321.23 ± 9.79 ^cd^
Acetic (2%)	144.62 ± 6.50 ^d^	53.73 ± 7.32 ^e^	64.47 ± 1.45 ^d^	8.74 ± 1.14 ^bcd^	357.85 ± 8.80 ^e^	257.98 ± 8.94 ^e^
Acetic (4%)	150.73 ± 3.88 ^d^	50.91 ± 2.47 ^e^	64.41 ± 7.63 ^d^	8.15 ± 0.47 ^cd^	358.59 ± 12.15 ^e^	286.66 ± 21.46 ^de^
Ascorbic (1%)	237.58 ± 9.18 ^a^	91.39 ± 2.15 ^a^	91.61 ± 3.65 ^a^	9.60 ± 0.67 ^bc^	541.77 ± 18.01 ^a^	428.63 ± 31.57 ^a^
Ascorbic (2%)	202.80 ± 5.13 ^b^	78.66 ± 3.75 ^bc^	76.16 ± 5.79 ^b^	9.94 ± 0.63 ^b^	462.12 ± 15.35 ^b^	371.71 ± 28.81 ^b^
Ascorbic (4%)	195.47 ± 4.91 ^b^	70.81 ± 3.76 ^cd^	61.29 ± 0.97 ^d^	8.85 ± 0.27 ^bcd^	431.37 ± 3.97 ^bc^	321.02 ± 1.28 ^cd^
Citric (1%)	172.16 ± 8.00 ^c^	74.06 ± 2.24 ^bcd^	66.42 ± 1.20 ^cd^	8.82 ± 0.54 ^bcd^	421.42 ± 3.14 ^cd^	300.17 ± 5.39 ^d^
Citric (2%)	192.81 ± 12.30 ^b^	64.47 ± 7.52 ^d^	58.45 ± 2.36 ^d^	7.35 ± 0.35 ^d^	417.87 ± 30.50 ^cd^	320.75 ± 28.09 ^cd^
Citric (4%)	202.57 ± 10.65 ^b^	67.71 ± 5.51 ^d^	60.86 ± 1.93 ^d^	8.10 ± 0.29 ^cd^	450.18 ± 9.63 ^bc^	348.67 ± 9.07 ^bc^
Fisher’s LSD **	15.86	10.22	9.73	1.53	32.36	39.91

* Mean and standard deviation of three replicates. ** For each column, superscript letters indicate statistical groupings based on Fisher’s least significant difference (LSD) test. Different letters denote statistically significant differences, while identical letters indicate no significant difference at the 95% confidence level.

**Table 5:** Volatile compounds (C5 and C6) from the lipoxygenase pathway and ethanol in oils *.

Acid	Total C6 LOX, mg/kg	Total C5 LOX, mg/kg	Ethanol, mg/kg
Control (0%)	8.69 ± 0.56 ^e^	2.01 ± 0.05 ^de^	4.56 ± 0.19 ^a^
Acetic (1%)	10.07 ± 0.62 ^d^	1.80 ± 0.00 ^e^	2.52 ± 0.10 ^c^
Acetic (2%)	7.15 ± 0.57 ^e^	1.43 ± 0.08 ^f^	2.30 ± 0.16 ^cd^
Acetic (4%)	4.67 ± 0.29 ^f^	0.49 ± 0.04 ^g^	1.93 ± 0.17 ^d^
Ascorbic (1%)	12.82 ± 0.61 ^c^	2.23 ± 0.11 ^d^	3.74 ± 0.53 ^b^
Ascorbic (2%)	10.92 ± 0.76 ^d^	3.03 ± 0.15 ^b^	2.81 ± 0.36 ^c^
Ascorbic (4%)	14.07 ± 1.13 ^b^	3.27 ± 0.03 ^ab^	4.33 ± 0.19 ^ab^
Citric (1%)	16.69 ± 0.34 ^a^	3.33 ± 0.29 ^a^	4.04 ± 0.48 ^ab^
Citric (2%)	15.09 ± 0.67 ^b^	2.09 ± 0.12 ^d^	4.23 ± 0.26 ^ab^
Citric (4%)	11.09 ± 0.64 ^d^	2.60 ± 0.12 ^c^	2.74 ± 0.24 ^c^
Fisher’s LSD **	1.06	0.22	0.52

* Mean and standard deviation of three replicates. ** For each column, superscript letters indicate statistical groupings based on Fisher’s least significant difference (LSD) test. Different letters denote statistically significant differences, while identical letters indicate no significant difference at the 95% confidence level.

## Data Availability

The original contributions presented in the study are included in the article/Supplementary Material, further inquiries can be directed to the corresponding author(s).

## Supplementary Materials

The following supporting information can be downloaded at https://www.mdpi.com/article/10.3390/foods14081336/s1, Table S1: Experimental design, Table S2: Phenolic acids and derivatives; Figure S1: Total phenolic and secoiridoid content and antioxidant capacity of olive oils.
