# Supplementary material for: Increasing the Bioactive Compound Content of Olive Oil by Acidification of Olive Paste
Source: Foods. 2025 Apr 12;14(8):1336. doi: 10.3390/foods14081336 (PMC12027470; doi:10.3390/foods14081336)
Supplement: Supplementary file 1 [file foods-14-01336-s001.zip › foods-3570491-supplementary.pdf]

Table S1. Experimental design

run	Acid	Dose, %
1	Citric (cit)	4
2	Acetic (act)	4
3	Ascorbic (asc)	2
4	Citric (cit)	4
5	Ascorbic (asc)	4
6	Ascorbic (asc)	1
7	Ascorbic (asc)	2
8	Citric (cit)	4
9	Acetic (act)	1
10	Acetic (act)	2
11	Citric (cit)	2
12	Ascorbic (asc)	4
13	Citric (cit)	2
14	Citric (cit)	1
15	Citric (cit)	1
16	Acetic (act)	1
17	Acetic (act)	2
18	Control (ctr)	0
19	Ascorbic (asc)	1
20	Citric (cit)	1
21	Acetic (act)	2
22	Ascorbic (asc)	1
23	Citric (cit)	2
24	Ascorbic (asc)	2
25	Control (ctr)	0
26	Acetic (act)	4
27	Control (ctr)	0
28	Acetic (act)	1
29	Ascorbic (asc)	4
30	Acetic (act)	4

Table S2. Phenolic acids and derivatives, mg/kg *

Acid	Vanillic acid	Vanillin	*p*-Coumaric acid	Ferulic acid
Control (0 %)	1.60 ± 0.11 [a]	0.12 ± 0.01 [a]	0.88 ± 0.07 [a]	0.35 ± 0.02 [ab]
Acetic (1 %)	2.62 ± 0.12 [de]	0.13 ± 0.01 [a]	1.36 ± 0.09 [bcd]	0.37 ± 0.04 [ab]
Acetic (2 %)	2.20 ± 0.33 [cd]	0.13 ± 0.01 [a]	1.34 ± 0.07 [bcd]	0.34 ± 0.02 [ab]
Acetic (4 %)	2.59 ± 0.25 [de]	0.10 ± 0.01 [a]	1.51 ± 0.16 [d]	0.39 ± 0.05 [ab]
Ascorbic (1 %)	1.74 ± 0.22 [ab]	0.64 ± 0.02 [b]	1.24 ± 0.08 [b]	0.92 ± 0.03 [d]
Ascorbic (2 %)	2.08 ± 0.04 [bc]	0.69 ± 0.06 [b]	1.30 ± 0.06 [bc]	0.86 ± 0.08 [d]
Ascorbic (4 %)	2.63 ± 0.37 [de]	0.66 ± 0.05 [b]	1.36 ± 0.10 [bcd]	0.73 ± 0.10 [c]
Citric (1 %)	2.99 ± 0.03 [e]	0.14 ± 0.01 [a]	1.33 ± 0.05 [bcd]	0.41 ± 0.03 [ab]
Citric (2 %)	2.28 ± 0.19 [cd]	0.11 ± 0.01 [a]	1.21 ± 0.16 [b]	0.32 ± 0.02 [a]
Citric (4 %)	2.32 ± 0.22 [cd]	0.12 ± 0.01 [a]	1.48 ± 0.03 [cd]	0.43 ± 0.03 [b]
Fisher's LSD **	0.44	0.06	0.20	0.10

* Mean and standard deviation of three replicates.

** For each column, superscript letters indicate statistical groupings based on Fisher's Least Significant Difference (LSD) test. Different letters denote statistically significant differences, while identical letters indicate no significant difference at the 95% confidence level.

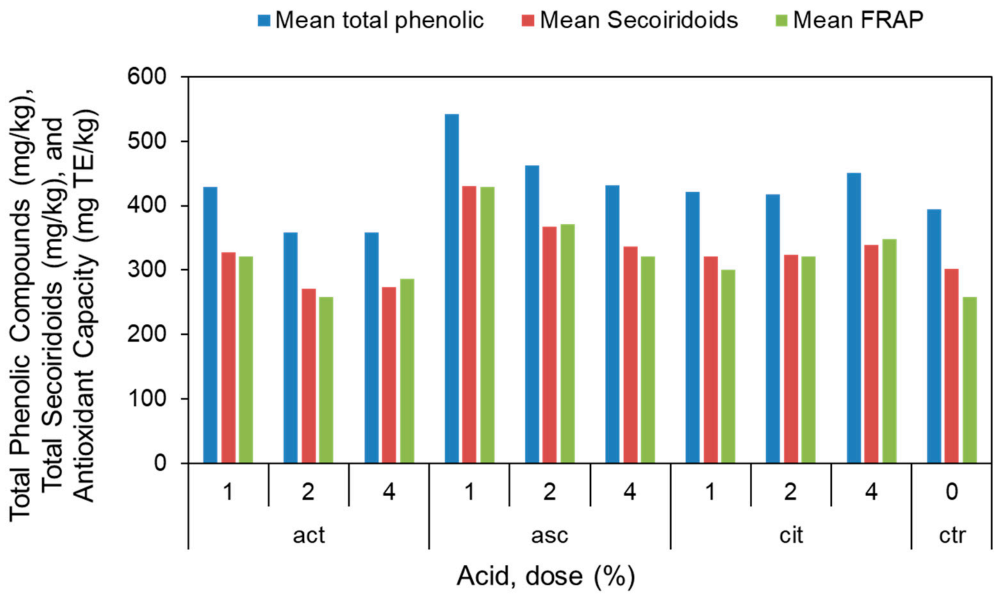

Figure S1. Total phenolic and secoiridoid content and antioxidant capacity of olive oils
